# Cardioprotective effects of naringin in a type 2 diabetes rodent model by reducing calcium overload and oxidative stress

**DOI:** 10.3389/fphar.2025.1621356

**Published:** 2025-08-06

**Authors:** Arkady Uryash, Alfredo Mijares, Jose Miguel Eltit, Jose A. Adams, Jose R. Lopez

**Affiliations:** ^1^ Division of Neonatology, Mount Sinai Medical Center, Miami, FL, United States; ^2^ Centro de Biofísica y Bioquímica, Instituto Venezolano de Investigaciones Científicas, Caracas, Venezuela; ^3^ Department of Physiology and Biophysics, Virginia Commonwealth University, Richmond, VA, United States; ^4^ Department of Research, Mount Sinai Medical Center, Miami, FL, United States

**Keywords:** cardiomyocytes, diabetes, naringin, calcium, diabetic cardiomyopathy, oxidative stress

## Abstract

**Introduction:**

Diabetic cardiomyopathy (DCM) is characterized by structural and functional alterations in the heart muscle, occurring independently of other cardiovascular risk factors such as dyslipidemia, hypertension, or coronary artery disease. Despite efforts to manage type 2 diabetes (T2D) and its complications, DCM remains a significant cause of morbidity and mortality in diabetic patients. The pathogenesis of DCM is multifactorial, involving oxidative stress, inflammation, and intracellular Ca^2+^ dyshomeostasis. Currently, there is no specific or effective treatment for DCM. Naringin (NRG), a flavonoid abundant in citrus fruits, has demonstrated promising cardioprotective properties.

**Methods:**

Cardiomyocytes were isolated from a 12-month-old murine T2D model (db/db mice) and corresponding age-matched control subjects. Naringin was administered via intraperitoneal injection at a dosage of 60 mg/kg for 4 weeks to evaluate its cardioprotective efficacy in DCM.

**Results:**

Quiescent cardiomyocytes from db/db mice showed significantly increased diastolic Ca^2+^ levels ([Ca^2+^]_d_), reactive oxygen species (ROS), lipid peroxidation, advanced oxidation protein products (AOPP), and nicotinamide adenine dinucleotide phosphate (NADPH) levels, along with reduced superoxide dismutase (SOD) activity and adiponectin (APN) levels. Plasma markers of cardiac injury were also elevated compared to those in the control group. NRG treatment significantly reduced [Ca^2+^]_d_, ROS, lipid peroxidation, AOPP, and NADPH levels while enhancing SOD activity and APN levels. Furthermore, NRG attenuated plasma cardiac injury markers in db/db mice.

**Conclusion:**

The results of this study illustrate the cardioprotective potential of NRG in diabetic cardiomyopathy by mitigating intracellular calcium overload and oxidative stress, augmenting antioxidant defenses, and reducing cardiac injury. NRG could serve as a promising adjunctive therapeutic approach to enhance cardiac function in diabetic patients.

## 1 Introduction

Diabetes mellitus (DM) constitutes a chronic, progressive metabolic disorder characterized by the dysregulation of blood glucose levels. The disorder is chiefly categorized into two primary subtypes: Type 1 diabetes mellitus (T1D) and Type 2 diabetes mellitus (T2D). T1D is attributed to autoimmune-mediated destruction of pancreatic β-cells, resulting in inadequate insulin production. Conversely, T2D primarily originates from insulin resistance in target tissues (Goyal and Mehta, 2013).

Diabetic cardiomyopathy (DCM) represents a noteworthy cardiovascular complication of DM, characterized by changes in myocardial structure and function, occurring independently of traditional cardiac risk factors such as coronary artery disease and hypertension (Rubler et al., 1972; Dillmann, 2019). Despite progress in the management of T2D and endeavors to avert its complications, DCM continues to be a significant contributor to morbidity and mortality among individuals with diabetes (Wussling et al., 1987). Numerous investigations indicate that diabetic patients face an elevated risk of heart failure, even when variables such as age, ethnicity, body mass index, dyslipidemia, and coronary artery disease are considered in comparisons with age-matched, non-diabetic controls (Wilson, 1998; Jankauskas et al., 2021).

Although hyperglycemia is a critical factor contributing to the progression of diabetic cardiomyopathy (DCM), evidence indicates that stringent glycemic management has not consistently demonstrated a reduction in morbidity or mortality rates among diabetic populations (Jia et al., 2018). Moreover, diabetes mellitus augments the myocardium’s vulnerability to ischemia-reperfusion injury and undermines the defensive mechanisms of cardiomyocytes against ischemic damage (Lejay et al., 2016). This highlights the inherent vulnerability of the diabetic heart, emphasizing the intricate relationship between diabetes and cardiovascular disease. It underscores the necessity for targeted therapeutic strategies that extend beyond mere glycemic control. Regrettably, current therapeutic interventions for DCM remain inadequate, thus highlighting the essential need for the development of innovative cardioprotective strategies aimed at alleviating heart failure and coronary disease in individuals suffering from diabetes.

Nutraceuticals, biologically active compounds derived from foods, offer a promising and innovative approach to promoting health and preventing the onset or progression of chronic diseases (McCarty, 2021). Among the nutraceuticals with notable therapeutic potential, naringin (NRG), a flavanone glycoside abundantly found in citrus fruits such as grapefruit, stands out for its broad spectrum of biological activities. Naringin has been shown to exert various pharmacological effects, including anticancer, anti-inflammatory, antioxidant, antidiabetic, anti-apoptotic, antiproliferative, and antimutagenic properties (Kwatra et al., 2016; Aoi et al., 2021; Luo et al., 2021; Varshney and Garabadu, 2021; Yaseen et al., 2024).

Naringin’s ability to modulate key signaling pathways involved in inflammation, oxidative stress, and antihyperglycemic effects is especially relevant to cardiovascular diseases (Lambev et al., 1980; Li et al., 2021; Li et al., 2023; Chen et al., 2024; Lu et al., 2024; Zhang et al., 2025). This is particularly important in conditions like DCM, where the dysregulation of these pathways, especially chronic inflammation and oxidative stress, drives disease onset and progression. Persistent myocardial inflammation plays a central role in the initiation and progression of DCM, triggering a cascade of maladaptive events, including disruptions in intracellular Ca^2+^ homeostasis, oxidative stress, pathological myocardial hypertrophy, increased cardiomyocyte apoptosis, interstitial fibrosis, and the development of heart failure (Uryash et al., 2021b; Nakamura et al., 2022; Ramesh et al., 2022). Naringin has demonstrated significant efficacy in attenuating the expression of key signaling molecules associated with the inflammatory response, including interleukin-6 (IL-6), interleukin-8 (IL-8), inducible nitric oxide synthase, tumor necrosis factor-alpha (TNF-α), and nuclear factor erythroid 2 (Nrf2), in various animal models of inflammation (Habauzit et al., 2011). Persistent hyperglycemia promotes the activation of nuclear factor kappa-B (NF-κB), which has been widely implicated in the pathophysiology of DCM (Fu et al., 2020). The activation of NF-κB leads to the release of downstream pro-inflammatory cytokines, including mediators such as TNF-α, IL-6, IL-8, and monocyte chemoattractant protein, which are overexpressed in diabetic hearts (Frati et al., 2017). NRG reduces the elevated levels of TNF-α, IL-6, and NF-κB present in the db/db myocardium (Uryash et al., 2021b).

Oxidative stress is a well-recognized contributor to DCM, via NF–κB–mediated pathways, leading to an increased intracellular production of reactive oxygen species (ROS) (Evans et al., 2003; Uryash et al., 2021b). Another important factor contributing to the beneficial effects of NRG is its robust antioxidant activity (Zhang et al., 2025). NRG exerts a powerful ability to neutralize ROS, which are typically elevated in diabetic hearts by inhibiting the NF-κB signaling pathway, which is markedly upregulated in diabetic hearts (He et al., 2022). In addition, NRG activates the expression of several antioxidant enzymes, including heme oxygenase-1, glutathione S-transferase, superoxide dismutase, and catalase (Kwatra et al., 2016; Uryash et al., 2021b). These effects collectively contribute to the reestablishment of redox balance, thereby protecting the diabetic heart from oxidative damage and functional deterioration.

Impaired intracellular Ca^2+^ handling is a well-established hallmark of DCM, contributing to both diastolic dysfunction and cardiomyocyte injury (Popescu et al., 2019; Uryash et al., 2021b). In the context, cardiomyocytes from the diabetic mouse model exhibit elevated diastolic Ca^2+^ levels, a pathological feature attributed mainly to the reduced activity of the sarcoplasmic/endoplasmic reticulum Ca^2^-ATPase 2a (SERCA2a) pump (Ganguly et al., 1983; Trost et al., 2002) and increased calcium leak of ryanodine receptors mediated by Ca^2^/calmodulin-dependent protein kinase II (CaMKII) (Federico et al., 2017; Benchoula et al., 2023). Perturbation in Ca^2+^ handling by the mitochondria has also been reported to play a role in the development of diabetic cardiomyopathy (Dia et al., 2020). Although previous studies have reported alterations in the Na^+^/Ca^2+^ exchanger (NCX) in streptozotocin - or alloxan-induced diabetic models (Golfman et al., 1998; Choi et al., 2002), no significant changes in NCX expression or function have been observed in cardiac cells from db/db mice (Al Kury, 2020).

More recently, it has been demonstrated that NRG lowers diastolic Ca^2+^ concentrations in a dose-dependent manner in cardiomyocytes isolated from a diabetic rodent model (Uryash et al., 2021b). This calcium-lowering effect appears to be mediated, at least in part, by the upregulation of ATP-sensitive potassium (K_ATP_) channels, which are commonly downregulated in diabetic hearts (Uryash et al., 2021b). It is well established that K_ATP_ channels act as metabolic sensors that stabilize cellular ATP/ADP balance and membrane excitability; their opening hyperpolarizes the membrane, shortening action potentials and reducing Ca^2+^ influx (Noma, 1983). Interestingly, NRG enhances the expression of K_ATP_ channel subunits (Kir6.2, SUR1, and SUR2), promoting cardioprotection in the context of diabetic cardiomyopathy (Uryash et al., 2021b).

In this investigation, we hypothesized that NRG confers cardioprotective effects in diabetic cardiomyopathy by mitigating oxidative stress and diminishing cell injury. Specifically, we aimed to examine whether NRG treatment could attenuate markers of oxidative damage, including lipid peroxidation, protein carbonyl content, nicotinamide adenine dinucleotide phosphate levels, and advanced oxidation protein products, while augmenting the activity of antioxidant enzymes such as superoxide dismutase. Additionally, the study aimed to evaluate whether NRG could increase plasma levels of adiponectin and decrease plasma injury markers, including lactate dehydrogenase and creatine kinase-MB isoenzyme in db/db mice.

## 2 Materials and methods

### 2.1 Murine model

Experiments were conducted in 12-month-old male and female non-diabetic C57BL/6J (control) mice and diabetic C57BL/KsJ-db/db mice. The C57BL/KsJ-db/db mice are characterized by their lack of functional leptin receptors and have been used as a model for T2D (Chen et al., 1996). db/db mice spontaneously develop hyperglycemia, glycosuria, and hyperinsulinemia, exhibiting phenotypic traits that closely resemble those of human T2D. All mice were maintained in a pathogen-free facility within the animal holding unit of the Harry Pearlman Biomedical Research Institute at Mount Sinai Medical Center (Miami Beach, FL, United States). Animals were housed in individually ventilated, transparent plastic cages for rodents, under standardized conditions (12-h light/dark cycle, temperature of 22°C ± 2°C, and relative humidity of 50%–60%). Food and water were provided *ad libitum*. All animal procedures were conducted in accordance with institutional guidelines and were approved by the Institutional Animal Care and Use Committee (IACUC), following the ethical principles outlined in the ARRIVE guidelines and the NIH Guide for the Care and Use of Laboratory Animals.

### 2.2 Study design

Control and db/db mice were divided into four groups. Group 1: Control mice, which did not receive NRG treatment but were administered distilled water intraperitoneally (IP) in a volume equivalent to that used for NRG injection. Group 2. Control mice were treated with NRG (60 mg/kg body weight, IP, once daily for 4 weeks), since this dose had the optimum effect on lowering [Ca^2+^]_d_ in db/db cardiomyocytes (Uryash et al., 2021b). Group 3. db/db mice, which did not receive NRG treatment; instead, they received an equivalent volume of distilled water as that used for NRG injection. Group 4. db/db mice treated with NRG (60 mg/kg body weight, IP, once daily for 4 weeks).

Body weights of control and db/db mice were recorded using an animal weighing scale (Kent Scientific Corporation, Torrington, CT, United States) at both the beginning and end of each of the four experimental protocols described above. To assess the diabetic phenotype and evaluate the potential hypoglycemic effect of NRG, fasting blood glucose levels were measured in both control and db/db mice at two time points: before the start of the protocol and after the 4-week treatment period, immediately before heart excision. Blood samples (5 µL) were collected from the tail vein following a 12-h fast. Glucose concentrations were determined using a glucometer (AlphaTRAK^®^ Glucose Meter, Abbott Animal Health, Abbott Park, IL, United States) as previously described (Andrikopoulos et al., 2008). Following the conclusion of the experimental protocols, mice received an intraperitoneal injection of heparin (1,000 U/kg) to prevent blood clot formation during subsequent perfusion and enzymatic digestion. The addition of heparin enhances the circulation of the enzyme solution within the myocardium, facilitating uniform tissue digestion and improving the efficiency of cardiomyocyte dissociation (Louch et al., 2011; Uryash et al., 2021b; Sedghi et al., 2023). Thirty minutes later, mice were anesthetized with ketamine (100 mg/kg) and xylazine (5 mg/kg) prior to scheduled heart excision.

### 2.3 Isolation of cardiomyocytes and inclusion criteria

The hearts of anesthetized mice were promptly excised, attached to a cannula, and affixed to a temperature-controlled system (maintained at 37°C). Following established protocols, cardiomyocytes from the left ventricle were enzymatically dissociated using the Langendorff retrograde coronary perfusion system (Uryash et al., 2021a; Uryash et al., 2021b). Only cardiomyocytes that did not contract upon exposure to a physiological solution containing Ca^2+^ and showed rod-shaped morphology, distinct borders, and well-defined striations were used for experiments within 1–5 h after isolation (Uryash et al., 2021b). We used freshly isolated cardiomyocytes to minimize dedifferentiation and changes in cellular function that occur when cells are cultured (Bird et al., 2003).

### 2.4 Simultaneous measurements of [Ca^2+^]_d_ and ROS production in cardiomyocytes

In quiescent cardiomyocytes from control and db/db mice [Ca^2+^]_d_ was measured using double-barreled Ca^2+^-selective microelectrodes, as previously reported (Eltit et al., 2010; Uryash et al., 2021b). The potential from the 3M KCl barrel (resting membrane potential) was electronically subtracted from that of the Ca^2+^-selective barrel (V_CaE_) to derive a Ca^2+^-specific potential, indicative of cardiomyocyte [Ca^2+^]_d_. Cardiomyocyte [Ca^2+^]_d_ measurements from control and db/db mice were deemed acceptable if they adhered to preestablished criteria: 1) a resting membrane potential more negative than −80 mV; 2) rapid stabilization of membrane potential (Vm) after microelectrode impalement; 3) stable recordings of Vm and Ca^2+^ specific potential (VCa_E_) for a minimum duration of 60 s; 4) prompt reversion to baseline of Vm and VCa_E_ upon electrode withdrawal. Data that did not meet these criteria were excluded from the analysis to ensure the consistency and reliability of the results. The intracellular generation of reactive oxygen species (ROS) was measured in quiescent cardiomyocytes using the cell-permeable compound 2′,7′-dichlorodihydrofluorescein diacetate (DCFH-DA) (Catalog# D6883, Sigma-Aldrich, MO, United States), which becomes fluorescent when oxidized by ROS inside the cell (DeAtley et al., 1999; Uryash et al., 2021b; Zhang et al., 2021).

Cardiomyocytes loaded with DCFH-DA from quiescent control and db/db mice were transferred to an experimental chamber and positioned on the stage of an inverted fluorescence microscope (Zeiss Axiovert 200, NY, United States). The cells were then impaled with a double-barreled Ca^2+^ microelectrode. DCF fluorescence was recorded using excitation and emission wavelengths of 480 nm and 535 nm, respectively. The rate of DCF fluorescence in all groups was normalized to the values obtained from untreated control cardiomyocytes. Experiments were carried out at 37.

### 2.5 Assessment of lipid peroxidation levels

Lipid peroxidation levels in cardiac tissue homogenates from both control and diabetic (db/db) mice, with and without naringin treatment, were evaluated by quantifying malondialdehyde (MDA) formation, a commonly used marker of lipid oxidative damage. MDA levels were measured using a commercially available colorimetric assay kit (Catalog #233471, Abcam, Waltham, MA, United States) according to the manufacturer’s protocol. To account for variations in protein concentration, MDA values were normalized to the total protein content of each sample and expressed as nanomoles per milligram of protein.

### 2.6 Measurement of AOPP levels

Left ventricular myocardial tissue samples from naringin-treated and untreated control and db/db mice were homogenized, and the supernatant was collected for the measurement of advanced oxidation protein products using the AOPP assay kit (Catalog #242295, Abcam, Waltham, MA, United States). This kit enables the detection of AOPP in plasma, lysates, and tissue homogenates. Oxidative stress was assessed by quantifying AOPP in left ventricular homogenates from both control and experimental groups, following the manufacturer’s instructions. Absorbance was measured at 340 nm using a microplate reader (Thermo Fisher Scientific, Waltham, MA, United States). Protein concentrations in each sample were expressed in mM.

### 2.7 Measurements of NADPH oxidase

NADPH oxidase activity was determined using the NADP/NADPH oxidase activity detection kit (Catalog #65349, Abcam, Waltham, MA, United States), following the manufacturer’s instructions. The assay quantifies the enzymatic conversion of NADP to NADPH, which is subsequently linked to a fluorescent signal. Fluorescence intensity was measured at 450 nm using a microplate reader (Thermo Fisher Scientific, Waltham, MA, United States). Fluorescence values of NADP/NADPH oxidase were normalized relative to those measured in untreated control groups.

### 2.8 Determination of SOD enzymatic activity

Left ventricular myocardial tissue samples were collected from both naringin-treated and untreated control and db/db mice. These samples were thoroughly homogenized to obtain tissue lysates for enzymatic analysis. Total superoxide dismutase (SOD) activity in the myocardial homogenates was measured using a commercially available colorimetric assay kit (Catalog #ab65354, Abcam, Cambridge, MA, United States), according to the manufacturer’s instructions. The absorbance of the dye was measured at 450 nm using a microplate reader (Thermo Fisher Scientific, Waltham, MA, United States). SOD activity was quantified and normalized to the total protein concentration in each sample. Results were expressed as units of SOD activity per milligram of protein (U/mg protein).

### 2.9 Measurement of plasma adiponectin

Blood was collected from untreated, NRG-treated control, and db/db mice. Mice were anesthetized with ketamine (100 mg/kg) and xylazine (5 mg/kg) to facilitate tail vein blood collection. Blood samples were subjected to centrifugation at 1,500 g for 15 min to isolate plasma, which was subsequently preserved at −70°C for further analysis. Plasma adiponectin (APN) levels were quantified using a commercial ELISA assay kit (Catalog # Ab226900, Abcam, Cambridge, MA, United States), according to the manufacturer’s guidelines.

### 2.10 Determination of cardiac markers in serum

At the conclusion of the experimental protocol, blood samples were collected via tail vein puncture from anesthetized mice (ketamine, 100 mg/kg, and xylazine, 5 mg/kg) to assess serum levels of lactate dehydrogenase (LDH) and creatine kinase-MB (CK-MB), two key biomarkers of cardiac injury. Cardiac puncture was deliberately avoided to prevent mechanical damage to the heart, which could artificially elevate the concentrations of these markers. LDH levels were quantified using a colorimetric LDH assay kit (Catalog #102526, Abcam, Waltham, MA, United States), while CK-MB levels were determined using a specific CK-MB assay kit (Catalog #285231, Abcam, Waltham, MA, United States), both according to the manufacturers’ instructions. Absorbance was measured at 450 nm using a microplate reader (Thermo Fisher Scientific, Waltham, MA, United States). Results are reported as international units per liter (IU/L).

### 2.11 Solutions

All solutions were prepared using ultrapure water sourced from a Milli-Q system (Millipore, Bedford, MA, United States). The composition of the Tyrode solution was as follows (in mM): NaCl, 130; KCl, 5; CaCl_2,_ 1.8; MgCl_2_, 1; glucose, 5; NaHCO_3,_ 20; and NaH_2_PO_4_, 0.9 (pH of 7.3). The solution was equilibrated with 95% O_2_ and 5% CO_2,_ and all experimental procedures were carried out at a temperature of 37°C.

### 2.12 Statistical analyses

Data are presented as mean ± standard deviation (SD). The normal distribution of the samples was evaluated utilizing the D’Agostino & Pearson test. For the analysis of the experimental data, a t-test as well as one-way and two-way analyses of variance (ANOVA) were used, followed by the Tukey multiple comparison test, to determine the statistical significance between the various groups. *n*_*mice*_ represents the total number of mice utilized in the study, while *n*_*cells*_ the quantity of successful measurements obtained from cardiomyocytes. A value of *p* < 0.05 was considered statistically significant.

## 3 Results

### 3.1 Effects of naringin on body weight and blood glucose in db/db mice

**FIGURE 1:**
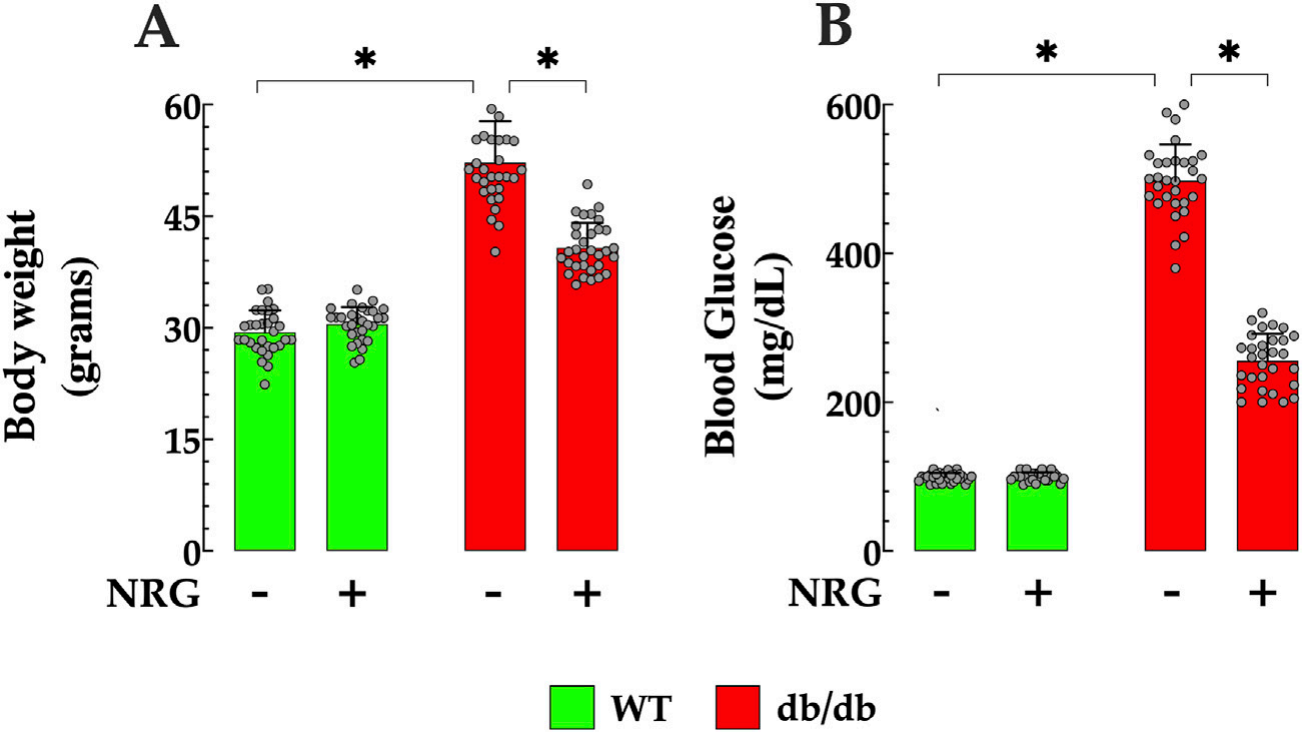
Naringin reduces body weight and fasting blood glucose in db/db mice. **(A)** Diabetic (db/db) mice exhibited significantly higher body weights, showing a 79% increase compared to controls. NRG treatment resulted in a 23% reduction in body weight in db/db mice compared to untreated db/db counterparts, with no significant changes observed in the control group. **(B)** Baseline fasting glucose levels were markedly elevated in db/db mice, 403% higher than in controls. Following treatment, NRG reduced glucose levels in db/db mice by 49% compared to untreated db/db mice, while glucose levels in control mice remained unchanged. Data are expressed as mean ± SD, with individual data points represented by grey circles (*n*_*mice*_ = 30 per group). *Indicate *p* < 0.05.

Body weight measurements were recorded in control and db/db mice before and after the completion of the 4-week experimental protocol. Prior to the initiation of the protocols, the average body weight of control mice was 29 ± 2.9 g (*n*_*mic*e_ = 60), while db/db mice exhibited significantly higher weights, averaging 52 ± 6 g (*n*_*mice*_ = 60, *p* < 0.05 compared to controls mice) (Figure 1A). Following NRG administration, db/db mice showed a significant reduction in body weight to 40 ± 3.3 g (*n*_*mice*_ = 30, *p* < 0.05 compared to NRG-untreated db/db mice) (Figure 1A). In contrast, no significant change was observed in control mice treated with NRG (30 ± 2.7 g, *n*_*mice*_ = 30, *p* = 0.06) (Figure 1A).

The fasting blood glucose levels in both control and db/db mice were assessed before and after the conclusion of the 4-week experimental protocol. The results revealed that the average fasting glucose level in the control group was 99 ± 6 mg/dL (*n* = 60) (Figure 1B). In contrast, db/db mice had markedly elevated levels as expected, averaging 498 ± 49 mg/dL (*n*_*mice*_ = 60, *p* < 0.05 compared to control mice). Following the 4 weeks, NRG-treated db/db mice showed a significant reduction in fasting glucose to 256 ± 36 mg/dL mg/dL (*n*_*mice*_ = 30, *p* < 0.05 compared to NRG-untreated db/db mice) (Figure 1B). In contrast, no significant change was observed in the control group after NRG treatment (100 ± 6 mg/dL, *n*_*mice*_ = 30, *p* = 0.22) compared to the NRG-untreated control mice) (Figure 1B). Fasting blood glucose levels in NRG-untreated db/db and control mice did not show statistically significant changes compared to their baseline values at the start of the protocol (*p* = 0.008 and *p* = 0.16, respectively) (Figure 1B).

### 3.2 Naringin attenuates [Ca^2+^]_d_ dysregulation and oxidative stress in db/db cardiomyocytes

Intracellular Ca^2+^ dysfunction and oxidative stress play a key role in the pathogenesis of diabetes (Dhalla et al., 2020). Prior investigations carried out by our laboratory have demonstrated that quiescent cardiomyocytes from db/db mice, which serve as a model for T2D, exhibit elevated [Ca^2+^]_d_ levels alongside increased intracellular ROS production (Uryash et al., 2021b). Since intracellular Ca^2+^ overload augments mitochondrial ROS production (da Silva et al., 2015), in the current study, we further examined the interrelationship between [Ca^2+^]_d_ and ROS production by simultaneously measuring both parameters in real-time from a single quiescent cardiomyocyte. Furthermore, we investigate the cardioprotective effects of naringin on individual cardiomyocytes from which [Ca^2+^]_d_ and ROS levels were measured simultaneously.

**FIGURE 2:**
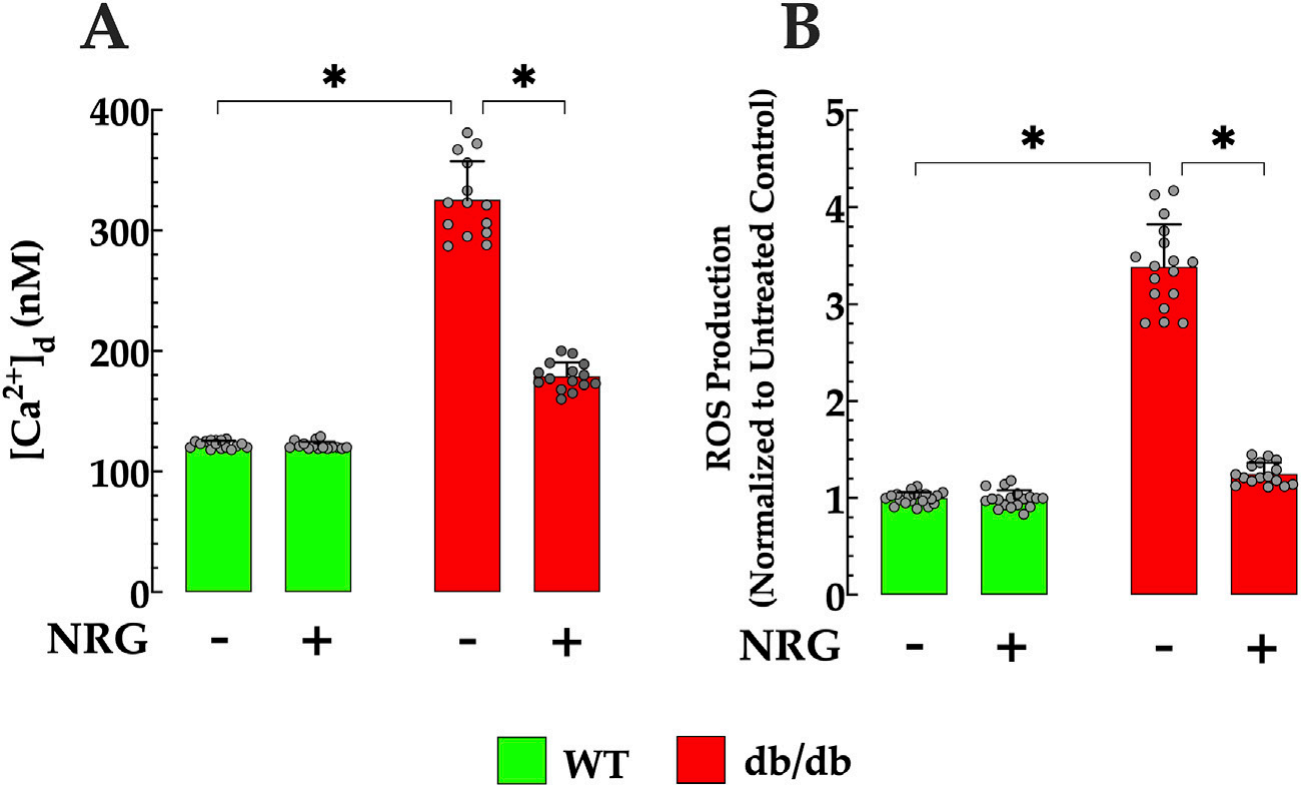
Effect of naringin on [Ca^2+^]_d_ and ROS generation in db/db cardiomyocytes. **(A)** Diastolic calcium concentration ([Ca^2+^]_d_) and **(B)** reactive oxygen species (ROS) production, measured simultaneously in individual cardiomyocytes, were significantly elevated in db/db mice compared to controls (by 167% and 241%, respectively). NRG treatment markedly reduced both [Ca^2+^]_d_ and ROS levels in db/db cardiomyocytes (by 45% and 63%, respectively), with no significant effects observed in control cells. Data are expressed as mean ± SD, with individual data points represented by grey circles (*n*_*mice*_ = 5 per group; *n* = 14–21). *Indicate *p* < 0.05.

In control quiescent cardiomyocytes, the mean [Ca^2+^]_d_ was 123 ± 3 nM, while in db/db cardiomyocytes, it was significantly elevated at 326 ± 33 nM (*p* < 0.05 compared to control cardiomyocytes) (Figure 2A). ROS production (fluorescence signal rate) in db/db cardiomyocytes was 241% higher relative to controls (*p* < 0.05) (Figure 2B). In subsequent experiments, the effect of NRG on [Ca^2+^]_d_ and ROS generation was explored in both genotypes. In db/db quiescent cardiomyocytes, NRG led to a reduction in [Ca^2+^]_d_ to 179 ± 12 (*p* < 0.05 compared to untreated db/db cardiomyocytes) (Figure 2A). Furthermore, NRG treatment substantially reduced oxidative stress, as evidenced by a significant decrease (63%) in ROS production in db/db cardiomyocytes (*p* < 0.05 compared to untreated db/db) (Figure 2B). However, NRG did not cause a significant effect on [Ca^2+^]_d_ and ROS production in control cardiomyocytes (*p* = 0.41 and *p =* 0.67, respectively, compared to untreated controls) (Figures 2A,B). The results suggest that NRG administration exerts cardioprotective properties by mitigating intracellular Ca^2+^ overload and reducing oxidative stress in db/db cardiomyocytes, which may contribute to improved cardiac function in db/db mice.

### 3.3 Naringin reduces myocardial lipid peroxidation in db/db mice

**FIGURE 3:**
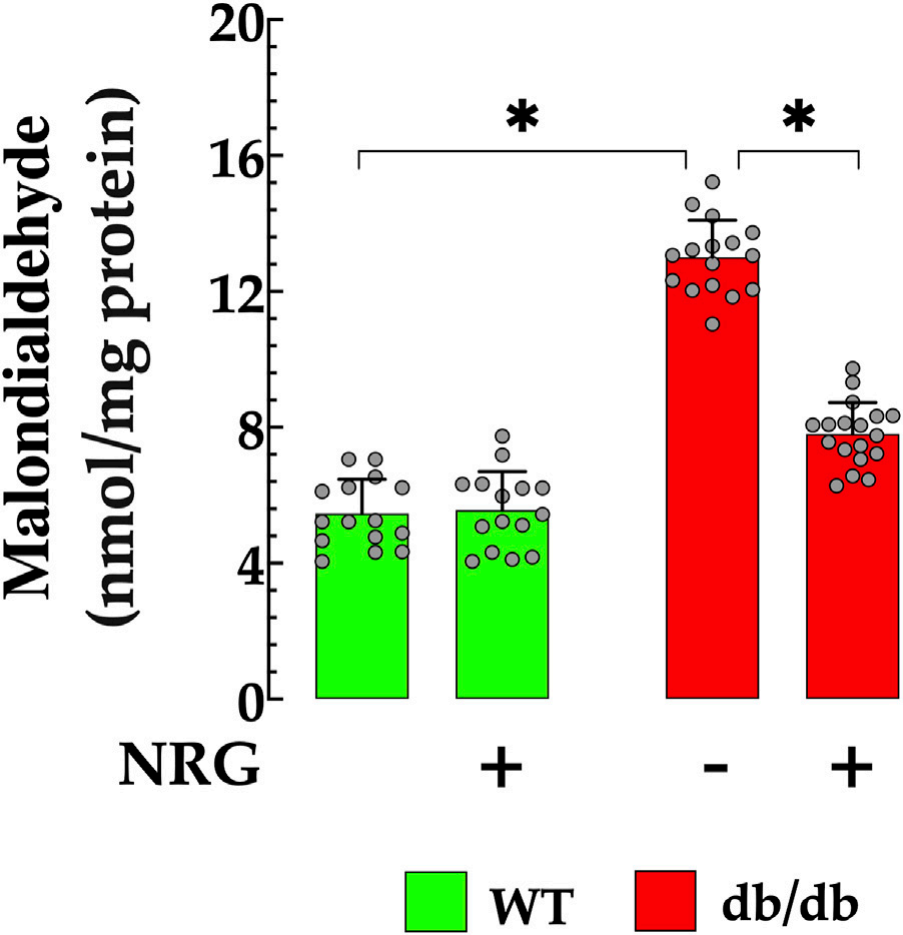
Naringin reduces lipid peroxidation in cardiac tissue of db/db mice. Lipid peroxidation, assessed by malondialdehyde (MDA) levels, was significantly elevated (by 116%) in db/db hearts compared to controls. NRG pretreatment reduced MDA levels by 46% in db/db cardiac tissue, with no significant effect observed in the control group. Data are presented as mean ± SD, with individual measurements shown as grey circles (*n*_*mice*_ = 6 per group; *n* = 15–18). *Indicate *p* < 0.05.

Increased lipid peroxidation, a well-established marker of oxidative damage, has been reported in the hearts of animal models with diabetic cardiomyopathy, including db/db mice (De Geest and Mishra, 2022; Peng et al., 2022). Oxidative stress was assessed by measuring malondialdehyde levels in cardiac tissue. Control tissues had MDA levels of 6 ± 0.99 nmol/mg protein, while db/db mice had significantly higher levels (13 ± 1.09 nmol/mg protein, *p* < 0.05), indicating pronounced oxidative stress (Figure 3). Pretreatment with NRG significantly reduced MDA levels in db/db cardiac tissue to 7 ± 0.93 nmol/mg protein (*p* < 0.05 compared to untreated db/db cardiac tissues), demonstrating NRG’s potential protective effect. In contrast, NRG had no significant effect on lipid peroxidation in control tissues (*p* = 0.79 compared to untreated control cardiac tissues) (Figure 3).

### 3.4 Naringin decreases myocardial AOPP accumulation in db/db mice

**FIGURE 4:**
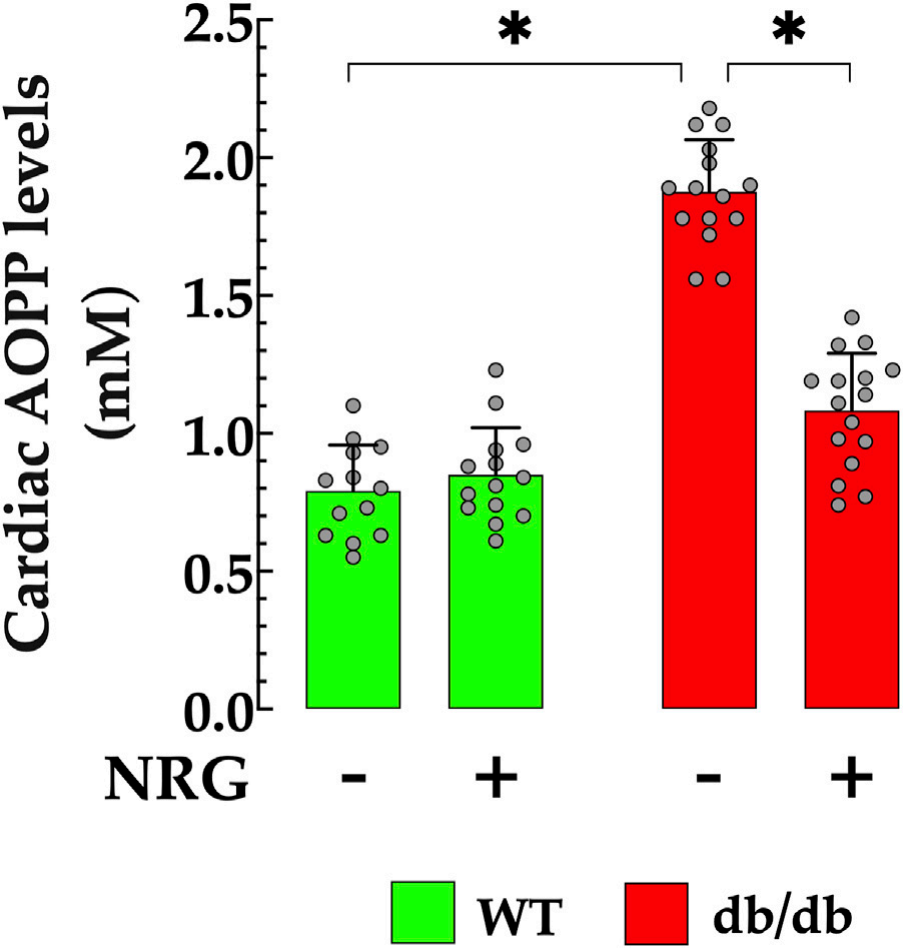
Reduction of AOPP levels by naringin in db/db cardiac tissue. Levels of advanced oxidation protein products (AOPPs) were significantly elevated (by 136%) in cardiac homogenates from db/db mice compared to controls. Pretreatment with NRG markedly reduced AOPP levels in db/db samples by 42%, while having no significant effect in control cardiac tissue. Data are presented as mean ± SD, with individual measurements shown as grey circles (*n*_*mice*_ = 6 per group; *n* = 14–16). *Indicate *p* < 0.05.

AOPP serves as an effective biomarker for assessing the extent of oxidative protein modifications, which are indicative of oxidative stress. Elevated serum concentrations of AOPP have been observed in patients with diabetic cardiomyopathy compared to healthy individuals (Kalousova et al., 2002; Conti et al., 2019). In db/db cardiac homogenates, AOPP was significantly elevated (1.87 ± 0.18 mM/mL) compared to the control group (0.79 ± 0.161 mM/L; *p* < 0.05) (Figure 4). Pretreatment with NRG markedly reduced AOPP content in db/db cardiac homogenates to 1.08 ± 0.21 mM/L (*p* < 0.05 vs. untreated db/db group), while it had no significant effect in control mice (*p* = 0.37 compared to NRG-untreated control) (Figure 4).

### 3.5 Naringin attenuated cardiomyocyte NADPH in db/db mice

**FIGURE 5:**
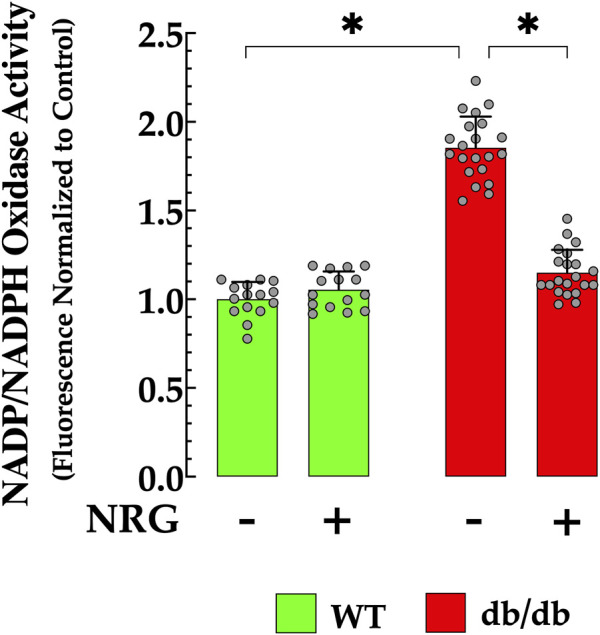
Naringin reduces elevated NADPH levels in db/db cardiac tissues. Nicotinamide adenine dinucleotide phosphate (NADPH) levels were markedly elevated in db/db cardiomyocytes (by 85%) compared to the control group. NRG treatment significantly reduced NADPH levels in db/db cardiac tissue (by 38% relative to the untreated group), with no notable effect observed in the control group. The data are reported as mean ± SD, with individual measurements represented as grey circles (*n*_*mice*_ = 4 per group; *n* = 15–21). *Indicate *p* < 0.05.

Previous studies have indicated NADPH plays a role in the adverse effects of cell damage induced by elevated glucose levels, and that increased concentrations have been correlated with oxidative stress in DCM (Gao and Mann, 2009; Xu et al., 2023). In the current study, the NADPH concentration was 85% higher in db/db cardiomyocytes compared to controls (*p* < 0.05) (Figure 5). Pretreatment with NRG significantly reduced NADPH by 38% in db/db cardiomyocytes compared to untreated db/db cardiomyocytes (*p* < 0.05), with no detectable effect on control *(p* = 0.15 compared to NRG-untreated control) (Figure 5).

### 3.6 Naringin enhances myocardial SOD activity in db/db mice

**FIGURE 6:**
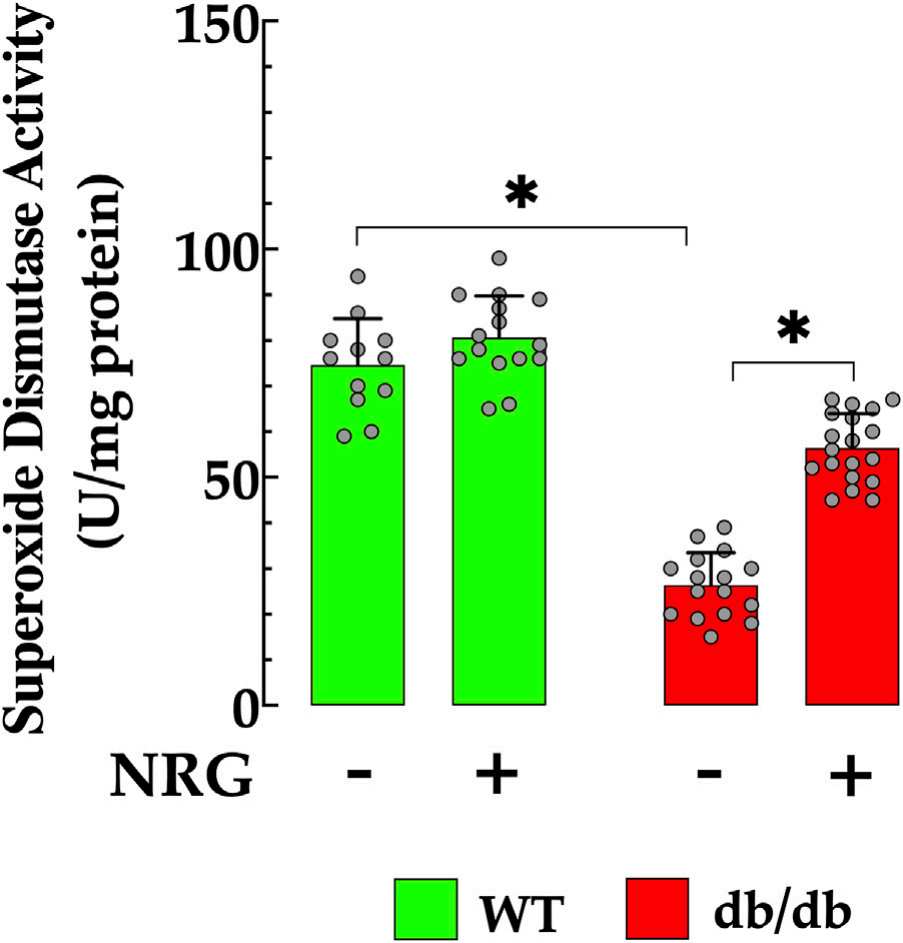
Enhancement of SOD activity induced by naringin in db/db cardiac tissues. Superoxide dismutase (SOD) activity was reduced by 88% in cardiac tissue from db/db mice compared to the control group. NRG administration enhanced SOD activity by 115% in db/db cardiac tissue, with no significant effect observed in the control group. Data are presented as mean ± SD, with individual measurements illustrated as grey circles (*n*_*mice*_ *= 6* per group and *n* = 12–19). *Indicate *p* < 0.05.

Oxidative stress plays a key role in attenuating antioxidant defenses, a process closely associated with the development of T2D (Promyos et al., 2023). In cardiac db/db homogenate, SOD activity was significantly diminished (26 ± 7.1 U/mg protein) when compared to those in control cardiac tissue (75 ± 10 U/mg protein, *p* < 0.05 relative to control tissue) (Figure 6). This reduction indicates an impaired antioxidant defense mechanism in diabetic cardiac tissues, rendering them more vulnerable to oxidative stress. Notably, administration of NRG in db/db mice resulted in a significant enhancement in SOD activity, reaching 56 ± 7.5 U/mg protein (*p* < 0.05 compared to untreated db/db mice) (Figure 6). The administration of NRG did not significantly alter the SOD levels in control cardiac cells (*p* = 0.93 compared to NRG-untreated control), suggesting that its positive effects are specifically directed towards the diabetic state (Figure 6).

### 3.7 Naringin lowers circulating adiponectin levels in db/db mice

**FIGURE 7:**
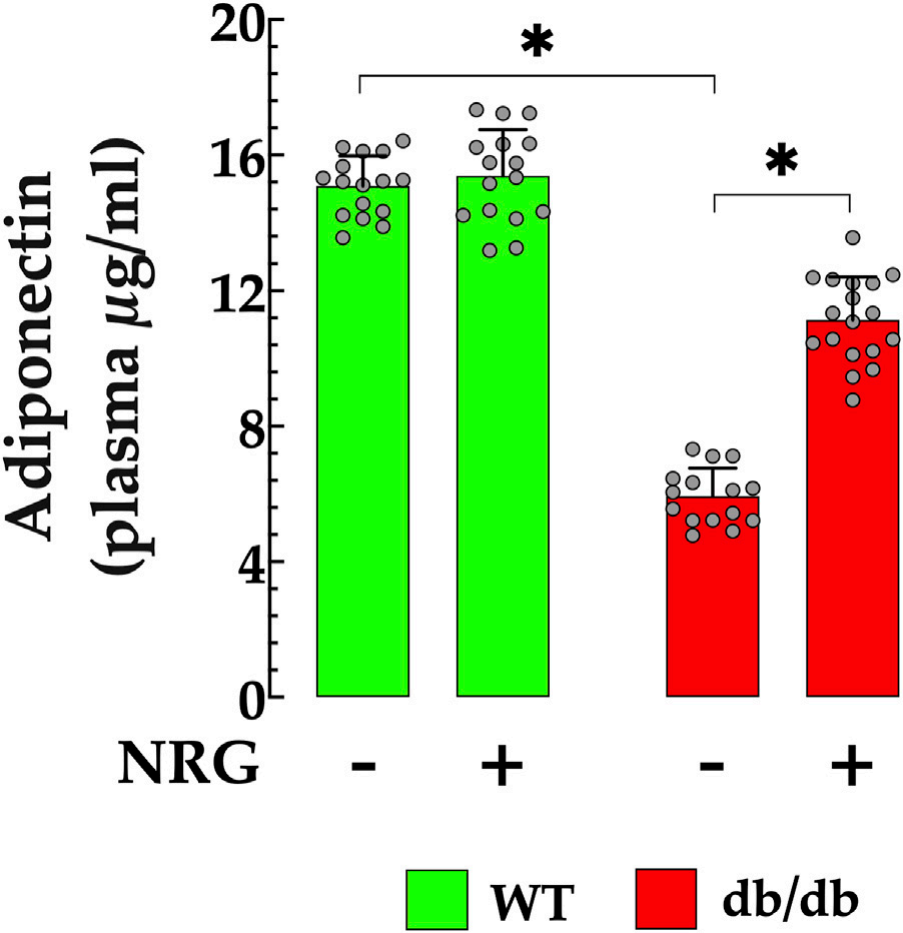
Naringin elevates plasma APN levels in db/db mice. Plasma adiponectin (APN) concentrations were markedly lower in db/db mice (by 60%) compared to the control group. NRG treatment significantly increased APN levels in db/db mice (by 83%), with no notable effect observed in the control group. Data are presented as mean ± SD, with individual measurements shown as grey circles (*n*_*mice*_ = 6 per group; *n* = 16–18). *Indicate *p* < 0.05.

Adiponectin is a well-established homeostatic factor that plays a critical role in regulating glucose levels and enhancing insulin sensitivity, primarily through its antioxidant and anti-inflammatory properties (Hong et al., 2023). In T2D patients with DCM, plasma APN levels are significantly lower compared to healthy individuals, indicating a strong association between low adiponectin levels and insulin resistance (Inoue et al., 2005). In db/db mice, plasma APN level was substantially lower 6 ± 0.82 ng/ml, compared to control mice, 15 ± 0.89 ng/ml (*p* < 0.05) (Figure 7). Nonetheless, the administration of NRG in db/db mice resulted in a significant elevation in plasma APN levels to 11 ± 1.3 ng/ml (*p* < 0.05 compared to untreated db/db mice) (Figure 7). The administration of NRG in control mice resulted in no statistically significant alteration in APN levels (*p* = 0.46 compared to NRG-untreated control), thereby affirming that its effects are specific to diabetic pathophysiological conditions.

### 3.8 Reduction of cardiac injury biomarkers in NRG-treated db/db mice

**FIGURE 8:**
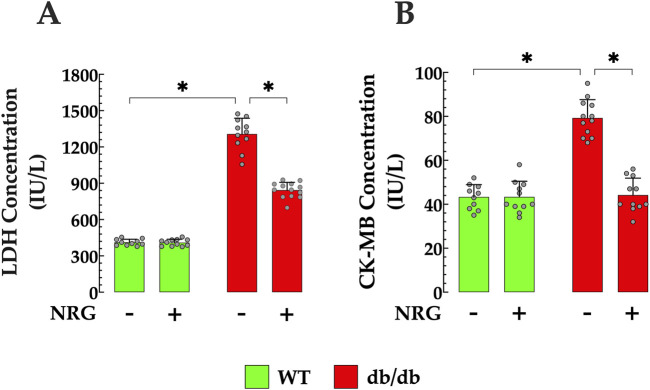
Naringin attenuates myocardial injury in diabetic myocardium. NRG administration reduced elevated serum levels of lactate dehydrogenase (LDH) by 35% and **(A)** creatine kinase-MB (CK-MB) by 44% in **(B)** db/db mice. Data are presented as mean ± SD, with individual measurements shown as grey circles (*n*_*mice*_ = 6 per group; *n* = 10–13). *Indicate *p* < 0.05.

Chronic hyperglycemia has been shown to elevate the risk of cardiac injury (Tan et al., 2020). DCM has been associated with elevated serum levels of LDH and CK-MB activity (Sun et al., 2014). db/db mice showed serum LDH levels that were significantly greater than those observed in the control group (411 ± 26 IU/L versus 1,307 ± 131 IU/L, *p* < 0.05 compared to control mice) (Figure 8A). Furthermore, in db/db mice, CK-MB levels were significantly elevated (79 ± 8 IU/L) compared to control mice (43 ± 5.65 IU/L, *p*<< 0.05) (Figure 8B). The administration of NRG significantly improved these cardiac injury markers in db/db mice, leading to a reduction in LDH levels to 843 ± 65 IU/L (*p* < 0.05 compared to untreated db/db mice) and CK-MB to 44 ± 7.7 IU/L (*p* < 0.05 compared to untreated db/db mice), without having statistically significant effects on the control group (*p* = 0.08 compared to NRG-untreated control) (Figures 8A,B).

## 4 Discussion

This study was conducted with the objective of examining the cardioprotective effects of NRG on diabetic cardiomyopathy. The principal novel findings of this research are.

• Ca^2+^ dysregulation and ROS production. db/db cardiomyocytes exhibited significantly elevated [Ca^2+^]_d_ and ROS levels than the control group. NRG treatment markedly reduced [Ca^2+^]_d_ and oxidative stress in db/db cardiomyocytes.
• Lipid peroxidation. Cardiac tissue from db/db models exhibits elevated lipid peroxidation levels compared to the control group, and this oxidative lipid damage was attenuated by NRG pretreatment.
• Advanced oxidation protein products. For the first time, it is demonstrated that the levels of AOPP are significantly elevated in cardiac tissue of db/db mice compared to the control group. Treatment with NRG notably decreased the aberrant levels of AOPP in the cardiac tissue of db/db mice.
• NADPH. NADPH was markedly elevated in db/db cardiomyocytes compared to the control group. The administration of NRG led to a significant reduction in NADPH levels in db/db cardiomyocytes compared to those not treated with NRG.
• Antioxidant defense. Superoxide dismutase activity was significantly decreased in db/db cardiac tissue compared to control cardiomyocytes. Treatment with NRG successfully reinstated SOD activity in db/db cardiac tissue compared to untreated tissue.
• Adiponectin levels. This study presents the first report showing that plasma adiponectin levels are lower in db/db mice compared to control mice. Furthermore, pretreatment with NRG significantly elevates APN levels in comparison to their untreated diabetic counterparts
• Cardiac injury markers. The db/db group demonstrated a marked elevation in LDH and CK-MB levels compared to the control group. The administration of NRG ameliorated these anomalies, reducing LDH and CK-MB levels in the db/db mice.

Diabetes mellitus represents a heterogeneous metabolic disorder characterized by persistent hyperglycemia, which results in compromised cellular functions concerning glucose transport and utilization. Over recent decades, heart disease has emerged as a leading cause of mortality and morbidity among diabetic patients. Within this context, diabetic cardiomyopathy has been increasingly recognized as a distinct pathological condition contributing to cardiac dysfunction in the diabetic population (Rubler et al., 1972; Aneja et al., 2008). DCM is characterized by structural and functional alterations of the myocardium in patients with diabetes mellitus, occurring independently of hypertension, coronary artery disease, or valvular pathology. These myocardial alterations lead to significant cardiac dysfunctions, including left ventricular hypertrophy, interstitial and perivascular fibrosis, intracellular lipid accumulation, and both diastolic and systolic failure. Collectively, these pathological changes play a crucial role in the progression of DCM, a condition whose underlying mechanisms, although not yet fully elucidated, are thought to involve disruptions in intracellular Ca^2+^ homeostasis, increased oxidative stress, chronic inflammation, lipotoxicity, and mitochondrial impairment (Ritchie and Abel, 2020; Uryash et al., 2021b). Despite its clinical relevance of DCM, no specific therapy has yet been identified to prevent or slow the progression of DCM (Nakamura et al., 2022). Effective management of hyperglycemia, through lifestyle modifications and lipid-lowering medications, is essential; however, DCM remains a persistent and challenging complication.

Naringin is a polyphenolic phytochemical commonly found in various herbs and fruits, such as oranges, lemons, grapes, grapefruit, bergamot, tomatoes, and cherries. NRG is easily soluble in water, and its absorption in the body is supported by the gut microbiota, which metabolizes naringin into an aglycone moiety (naringenin). NRG demonstrates potent antioxidant properties by scavenging free radicals and enhancing the activity of endogenous antioxidant enzymes, including superoxide dismutase and catalase (Oteiza et al., 2021; Zhao and Liu, 2021). Given these properties, NRG has emerged as a promising candidate for mitigating oxidative stress, which plays a critical role in the development and pathophysiology of DCM (Darenskaya et al., 2021; Jubaidi et al., 2021; Xu et al., 2023). In our previous work -now further supported by findings in the present study-we showed that NRG reduced hyperglycemia and protects db/db cardiomyocytes by reducing diastolic [Ca^2+^] overload and ROS intracellular production. Additionally, NRG enhanced glucose transport, suppressed myocardial inflammation, reduced calpain activity, improved cell viability, and restored the expression of K_ATP_ channel subunits Kir6.2, SUR1, and SUR2 (Uryash et al., 2021b).

Ca^2+^ ions serve as pivotal intracellular second messengers, playing an integral role in numerous cellular functions, such as cell survival and apoptosis, muscle contraction, metabolic regulation, and gene expression (Bkaily and Jacques, 2023). In quiescent excitable cells, cytosolic [Ca^2+^] is sustained at notably low levels, approximately 100 nM, whereas the extracellular milieu generally exhibits [Ca^2+^] exceeding 1 mM (Lopez et al., 1983; Uryash et al., 2021b; Uryash et al., 2024). The maintenance of low cytosolic [Ca^2+^] is accomplished through a meticulous equilibrium between Ca^2+^ influx and efflux mediated by ion channels, active transport, and ion exchanger mechanisms, respectively (Morciano et al., 2022). This finely tuned intracellular Ca^2+^ homeostasis not only preserves cytosolic Ca^2+^ balance but also orchestrates mitochondrial Ca^2+^ dynamics, which play a pivotal role in cellular metabolism and signaling (Matuz-Mares et al., 2022). Under physiological conditions and within a tissue-specific framework, the uptake of mitochondrial Ca^2+^ modulates the functionality of Krebs cycle enzymes and the activity of the electron transport chain, thereby eliciting the generation of ROS signaling (Matuz-Mares et al., 2022). This signaling axis operates within physiological [Ca^2+^]_d_ ranges; however, when these concentrations surpass the established threshold, mitochondrial ROS (mROS) production transitions from a regulatory role to a detrimental one, thereby disrupting mitochondrial bioenergetics and compromising cellular function (Gorlach et al., 2015). Mitochondrial Ca^2+^ overload can promote mROS formation both directly, by stimulating mROS-generating enzymes such as glycerol phosphate and α-ketoglutarate dehydrogenase, and indirectly, as evidenced by the activation of nitric oxide synthase, which produces nitric oxide and inhibits complex IV, leading to excessive mROS formation (Xu et al., 2020). Furthermore, mitochondrial Ca^2+^ overload precipitates the opening of the mitochondrial permeability transition pore, leading to mitochondrial membrane depolarization, uncoupling of oxidative phosphorylation, and ultimately, cell apoptosis (Hamilton et al., 2020).

The pathogenesis of DCM is acknowledged as a multifactorial process, with a growing body of evidence suggesting that perturbations in intracellular [Ca^2+^] homeostasis are intricately linked to the compromised mechanical function of the diabetic myocardium (Lebeche et al., 2008; Uryash et al., 2021b). Dysregulation of intracellular Ca^2+^ in diabetic cardiomyopathy is closely tied to impairments in key Ca^2+^ transport systems, which further exacerbate the mechanical deficits observed in the diabetic heart. At the cellular level, diabetic cardiomyopathy is marked by disrupted intracellular Ca^2+^ regulation, a well-established hallmark of DCM that plays a critical role in the diastolic dysfunction and cardiomyocyte injury (Uryash et al., 2021b). In this context, cardiomyocytes from diabetic mouse models—such as the db/db mouse—exhibit abnormally elevated diastolic Ca^2+^ levels, a pathological feature primarily linked to reduced activity of the sarcoplasmic/endoplasmic reticulum Ca^2+^-ATPase 2a (Ganguly et al., 1983; Trost et al., 2002) and increased calcium leak of ryanodine receptors mediated by Ca^2^/calmodulin-dependent protein kinase II (CaMKII) (Federico et al., 2017; Benchoula et al., 2023). While changes in the Na^+^/Ca^2+^ exchanger (NCX) have been noted in streptozotocin- or alloxan-induced diabetic models (Golfman et al., 1998; Choi et al., 2002), no significant changes in NCX expression or function are observed in db/db mouse cardiac cells (Al Kury, 2020).

Our prior investigation (Uryash et al., 2021b), which the current study has corroborated, identified an increase in both [Ca^2+^]_d_ and intracellular reactive oxygen species (ROS) production in cardiomyocytes of db/db mice compared to control cardiomyocytes. The enhanced production of ROS results in oxidative stress, leading to the generation and accumulation of oxidatively modified lipids, proteins, carbohydrates, and nucleic acids, which ultimately cause tissue damage (Choi et al., 2008). Excessive [Ca^2+^]_d_ and ROS damage proteins, nucleic acids, and lipids while also disrupting cellular antioxidant defenses, impacting apoptosis-related signaling pathways (Madreiter-Sokolowski et al., 2020). As previously reported by our group, NRG treatment significantly reduced hyperglycemia [Ca^2+^]_d,_ and intracellular ROS production in db/db cardiomyocytes compared to untreated db/db controls (Uryash et al., 2021b). NRG reduction of [Ca^2+^]_d_ and ROS production may be attributed to its effect on K_ATP_ channels expression and its ability to neutralize free radicals and boost endogenous antioxidant enzymes, respectively (Chen et al., 2015; Uryash et al., 2021b). The findings of the current study substantiate the interaction between [Ca^2+^]_d_ levels and ROS production, as evidenced by the reduction in ROS production achieved through modulating [Ca^2+^]_d_ overload in db/db cardiomyocytes using pharmacological and non-pharmacological interventions (Uryash et al., 2023; Uryash et al., 2024). This suggests that the dysregulation of [Ca^2+^]_d_ is linked to an elevation in ROS production in db/db cardiomyocytes, a mechanism that has been proposed as a potential cause of cardiac dysfunction in DCM (Uryash et al., 2021b). The antioxidant properties of NRG found in this investigation may be attributed to its ability to restore the protein expression of the Kir6.2, SUR1, and SUR2 subunits of the K_ATP_ channel at the sarcolemma (Uryash et al., 2021b)and subsequently induce membrane hyperpolarization, shortening of the action potential, and reduced Ca^2+^ influx, thereby preventing intracellular Ca^2+^ overload (Shimizu and Calvert, 2016). These findings highlight the therapeutic potential of NRG in counteracting intracellular [Ca^2+^] disruptions and atypical ROS generation under diabetic conditions.

Lipid peroxidation is a key feature of oxidative stress, particularly in metabolic disorders such as T2D (Davi et al., 2005). It occurs when ROS attacks polyunsaturated fatty acids in cellular membranes, triggering a chain reaction that compromises membrane integrity and cardiomyocyte function. In this context, our study reveals that db/db mice exhibit significantly elevated markers of lipid peroxidation compared to their control counterparts, indicating heightened oxidative stress and widespread lipid damage. Notably, NRG treatment markedly reduced hyperglycemia and lipid peroxidation in db/db mice, as evidenced by decreased MDA levels. By protecting lipid membranes from oxidative damage, NRG helps preserve cardiomyocyte integrity and function—especially crucial in the heart, a tissue highly susceptible to oxidative stress in diabetes. The observed reduction in lipid peroxidation following NRG treatment underlines its potential to mitigate oxidative stress and the resulting inflammatory responses triggered by peroxidation byproducts. Interestingly, NRG did not reduce lipid peroxidation in control mice, suggesting that its antioxidative effects are more pronounced under conditions of elevated oxidative stress, such as those found in diabetes. In healthy tissues with balanced redox homeostasis, NRG’s impact on lipid peroxidation appears minimal, highlighting its selective protective action in pathological states where oxidative stress is heightened. By reducing oxidative stress through dual mechanisms -scavenging ROS and decreasing lipid peroxidation- NRG emerges as a promising therapeutic candidate for use as an adjuvant in T2D treatment.

Increased concentrations of NADPH within db/db cardiomyocytes serve as an essential indicator of elevated oxidative stress, highlighting the common dysregulation of the redox balance associated with T2D. Our findings are in agreement with a recent report by Jin et al. (2023), which showed that db/db mice exhibit significantly elevated NADPH levels compared to control mice, indicating heightened oxidative stress and increased activity of NADPH oxidase, an enzyme complex responsible for generating superoxide, an important source of ROS. Remarkably, NRG treatment led to a reduction in hyperglycemia and significantly decreased NADPH levels in db/db cardiomyocytes. This reduction suggests that NRG mitigates the harmful effects of oxidative stress by influencing the NADPH oxidase pathway, which is often hyperactive in diabetic conditions (Vilas-Boas et al., 2021). By attenuating this pathway, NRG helps to curb excessive ROS production, thereby protecting the heart from oxidative damage, a critical factor in the development of diabetic cardiomyopathy and other cardiovascular complications associated with diabetes.

Advanced oxidation protein products are formed under conditions of chronic oxidative stress through reactions between plasma proteins and chlorinated oxidants, serving as markers of oxidative damage (Witko-Sarsat et al., 1996). AOPPs elevated values are correlated with insulin resistance and the presence or severity of diabetic complications (Heidari et al., 2020). This study demonstrates for the first time that AOPP levels are significantly higher in cardiomyocytes from db/db mice compared to controls, providing further evidence of the pronounced oxidative stress present in 12-month-old db/db mice. It is important to note that in this study, AOPP levels were measured in cardiac tissue rather than plasma, as plasma concentrations are often overestimated due to lipid interference (Heidari et al., 2020). In other tissues, such as podocytes, elevated levels of AOPPs have been shown to induce the generation of intracellular superoxide through the activation of NADPH oxidase, leading to increased caspase-3 activity and subsequent apoptosis (Zhou et al., 2009). Given the analogous oxidative stress pathways observed across diverse cell types, it is plausible to conjecture that a comparable mechanism operates in cardiac cells. Thus, AOPP-induced activation of NADPH oxidase may result in enhanced superoxide production, which subsequently triggers downstream signaling cascades that promote caspase-3 activation and apoptosis, ultimately culminating in cardiomyocyte death. NRG significantly reduced decreased hyperglycemia and AOPP levels, confirming its effectiveness in alleviating oxidative damage. This reduction highlights NRG’s ability to decrease protein oxidation and protect against sustained oxidative stress, which can otherwise lead to cardiomyocyte death and, ultimately, cardiac failure. Collectively, the decreases in AOPP levels accentuate NRG’s comprehensive approach to addressing oxidative stress: it not only impedes ROS generation by modulating NADPH oxidase activity but also diminishes oxidative protein damage.

In conditions characterized by sustained oxidative stress, as observed in T2D, a decrease in superoxide dismutase levels, an essential antioxidant enzyme, has been documented (You et al., 2016) which correlates with subsequent cardiac damage (Wang et al., 2020). In the present study, SOD levels were significantly reduced in db/db cardiomyocytes compared to control cells, consistent with previous findings in cardiomyocytes from streptozotocin-induced diabetic rats (You et al., 2016). Moreover, administration of NRG decreased hyperglycemia and markedly increased SOD activity in db/db myocardial tissue. Thus, NRG appears to protect the myocardial tissue of db/db mice by alleviating oxidative stress, both through the direct scavenging of free radicals (Cavia-Saiz et al., 2010) and by enhancing the endogenous antioxidant defense system. This is achieved, in part, through the upregulation of key antioxidant enzymes such as SOD, as demonstrated in the present study. In contrast, NRG did not notably affect SOD activity in control myocardial tissue, suggesting that the efficacy of NRG is more pronounced in pathological states characterized by oxidative imbalance, such as T2D.

Adiponectin, an adipose tissue-derived insulin sensitizer, is a key component of the interrelationship between adiposity and insulin resistance and is a significant risk factor for T2D (Silva et al., 2014). A meta-analysis of 15 prospective studies suggested that higher adiponectin levels were associated with a lower risk of diabetes across diverse populations (Li et al., 2009). Reduced plasma adiponectin levels have been consistently reported in patients with T2D, highlighting its association with insulin resistance and hypertension (Stojanovic et al., 2020). Similarly, significantly lower adiponectin levels have been observed in T2D rats, further supporting the relevance of adiponectin deficiency in the pathophysiology of diabetes-related complications (Gupta et al., 2020). This reduction in adiponectin may contribute to impaired glucose homeostasis, chronic inflammation, and increased cardiovascular risk observed in both human and experimental diabetes.

Notably, NRG significantly increases APN levels in db/db mice. To the best of our knowledge, this is the first study to demonstrate that pretreatment with NRG elevates APN levels in this diabetic model. This elevation may play a critical role in preventing or mitigating the progression of diabetes-related complications, including DCM. Although the precise mechanisms by which NRG modulates APN levels remain unclear, it is plausible that its hypoglycemic properties contribute to this effect. This is supported by evidence that insulin resistance suppresses APN production in various tissues, including adipocytes and skeletal muscle (Fang and Judd, 2018). Given the harmful metabolic and cardiovascular consequences associated with adiponectin deficiency, therapeutic strategies aimed at restoring APN levels—such as NRG supplementation—may offer significant clinical benefit.

Myocardial enzymes serve as critical biomarkers of cardiac injury, as they are released into the bloodstream when heart muscle cells undergo damage. These enzymes play a pivotal role in the early detection and diagnosis of myocardial damage. Numerous studies have demonstrated that serum levels of LDH, CK-MB, and aspartate aminotransferase are significantly elevated in conditions such as DCM (Li et al., 2018), reflecting myocardial stress and injury. In the present study, serum LDH and CK-MB levels were markedly elevated in the db/db group compared to the control group, indicating substantial cardiac damage. However, treatment with NGR effectively attenuated this increase in both biomarkers, suggesting a protective effect against myocardial injury. The cardioprotective effects observed with NGR in preserving myocardial integrity may be ascribed to its capacity to reduce [Ca^2+^]d overload and mitigate oxidative stress via the upregulation of KATP channels, which are acknowledged to fulfill a cardioprotective function (Niwano et al., 2012).

## 5 Conclusions

**FIGURE 9:**
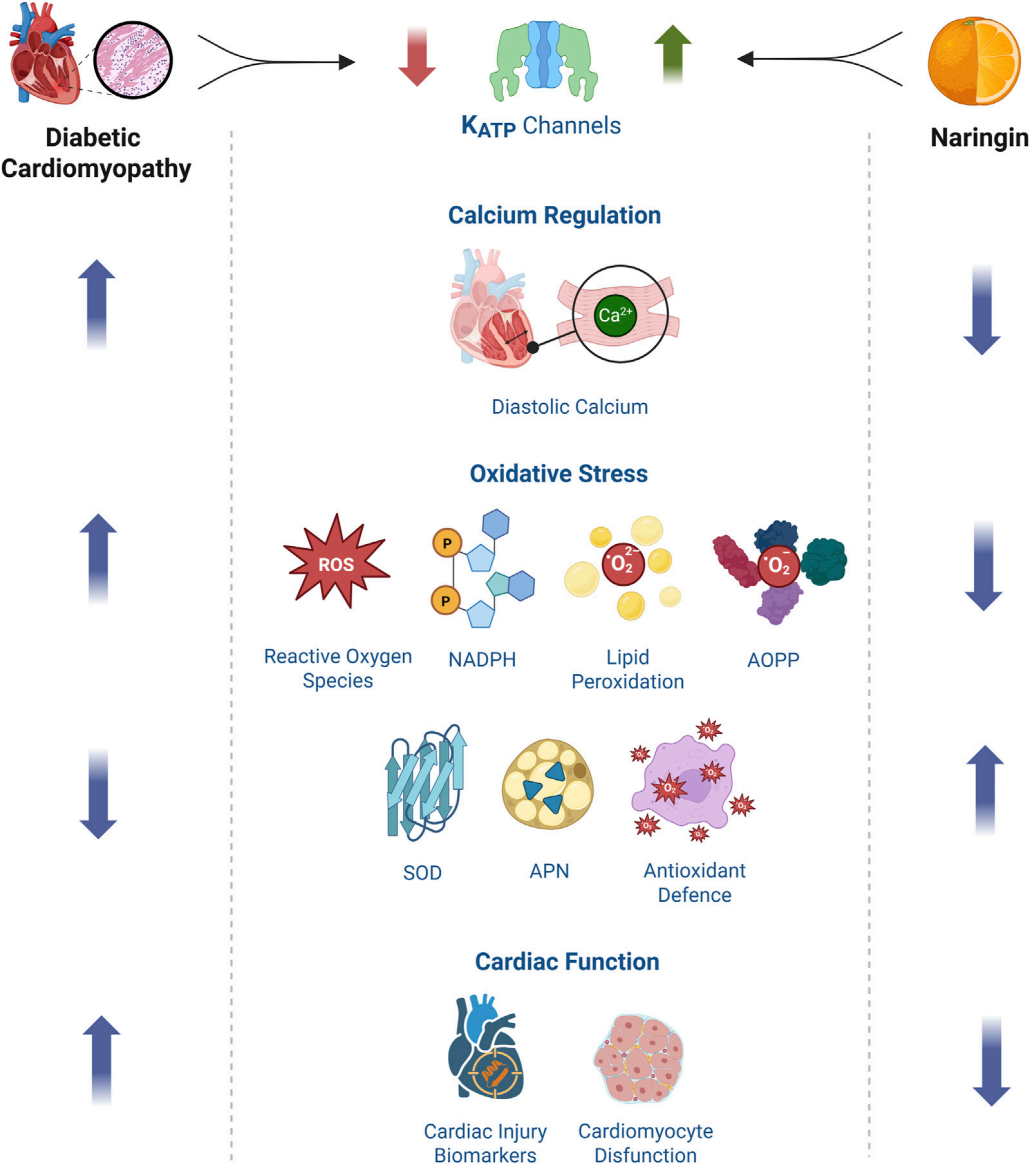
Proposed Mechanism of Naringin-Mediated Cardiac Protection. db/db cardiomyocytes displayed elevated diastolic [Ca^2+^]_d_, excessive production of ROS, increased lipid peroxidation, accumulation of AOPP, and elevated levels of NADPH, all indicative of heightened oxidative stress. Additionally, these mice exhibited reduced SOD activity and lower plasma APN levels, reflecting compromised antioxidant defenses and metabolic dysregulation. Furthermore, plasma biomarkers of cardiac injury were also significantly elevated, signaling myocardial damage. Administration of NRG markedly reduced [Ca^2+^]_d_, ROS production, lipid peroxidation, AOPP accumulation, and NADPH levels, indicating a substantial attenuation of oxidative stress. In parallel, NRG enhanced endogenous antioxidant defenses by upregulating SOD activity and restoring circulating APN concentrations. These molecular improvements were accompanied by a significant reduction in plasma cardiac injury markers, suggesting the preservation of myocardial structure and function. Mechanistically, the cardioprotective effects of NRG may be mediated, at least in part, through the upregulation of sarcolemmal K_ATP_ channels. Through the upregulation of the expression and/or activity of these channels, NRG facilitates the reduction of intracellular calcium overload and limits ROS generation, thereby disrupting a core pathophysiological mechanism that perpetuates oxidative stress and cardiac dysfunction in DCM.

This study provides novel insights into the cardioprotective effects of NRG in the context of DCM. Our findings demonstrate that NRG significantly reduces diastolic calcium levels, suppresses the generation of ROS, and attenuates lipid peroxidation, AOPP, and NADPH levels in the diabetic myocardium. These effects are accompanied by enhanced antioxidant defenses, particularly through the upregulation of SOD. Notably, to the best of our knowledge, this is the first comprehensive investigation to show that NRG elevates APN levels, a biomarker inversely associated with insulin resistance and cardiovascular disease, and concurrently reduces myocardial AOPP content, a marker of sustained protein oxidation and cellular injury. Collectively, these findings highlight the multifaceted cardioprotective actions of NRG (Figure 9) and support its potential as a therapeutic candidate for preventing or mitigating DCM by targeting both calcium dysregulation and oxidative stress pathways.

### 5.1 Study limitations

While this study provides compelling evidence for the cardioprotective effects of naringin in the context of DCM, several limitations should be acknowledged: 1) Age Considerations: Only 12-month-old db/db mice were used in the experiments. Given the known influence of age on DCM pathophysiology, additional studies across different age groups are necessary to confirm the generalizability of the observed protective effects. 2) Treatment duration: The study focused on molecular endpoints at a single time point following NRG treatment. Long-term studies are necessary to determine whether the observed molecular and biochemical changes result in sustained improvements in cardiac function and survival. 3) Lack of Functional Cardiac Assessments: Although biochemical and cellular markers of injury and oxidative stress were assessed, the study did not include *in vivo* cardiac function measurements (e.g., echocardiography, left ventricle hemodynamic parameters) to confirm the structural and functional impact of NRG on the diabetic heart.

## Data Availability

The original contributions presented in the study are included in the article/supplementary material, further inquiries can be directed to the corresponding author.
